# The impact of technology systems and level of support in digital mental health interventions: a secondary meta-analysis

**DOI:** 10.1186/s13643-023-02241-1

**Published:** 2023-05-04

**Authors:** Maxime Sasseville, Annie LeBlanc, Jack Tchuente, Mylène Boucher, Michèle Dugas, Mbemba Gisèle, Romina Barony, Maud-Christine Chouinard, Marianne Beaulieu, Nicolas Beaudet, Becky Skidmore, Pascale Cholette, Christine Aspiros, Alain Larouche, Guylaine Chabot, Marie-Pierre Gagnon

**Affiliations:** 1 Université Laval-VITAM Research Center on Sustainable Health, Quebec City, Canada; 2 VITAM Research Center on Sustainable Health, Quebec City, Canada; 3 Université Laval, Quebec City, Canada; 4 Université de Montréal, Montréal, Canada; 5 Université de Sherbrooke, Omnimed, Sherbrooke, Quebec Canada; 6 Centre Intégré Universitaire de Santé et de Services Sociaux de la Capitale Nationale, Quebec City, Canada

**Keywords:** Digital interventions, Mental health, Chronic diseases, Meta-analysis, Rapid review

## Abstract

**Background:**

The majority of people with a chronic disease (e.g., diabetes, hypertension, COPD) have more than one concurrent condition and are also at higher risk for developing comorbidities in mental health, including anxiety and depression. There is an urgent need for more relevant and accurate data on digital interventions in this area to prepare for an increase demand for mental health services. The aim of this study was to conduct a meta-analysis of the digital mental health interventions for people with comorbid physical and mental chronic diseases to compare the effect of technology systems and level of support.

**Methods:**

This secondary meta-analysis follows a rapid review of systematic reviews, a virtual workshop with knowledge users to identify research questions and a modified Delphi study to guide research methods: What types of digital health interventions (according to a recognized categorization) are the most effective for the management of concomitant mental health and chronic disease conditions in adults? We conducted a secondary analysis of the primary studies identified in the rapid review. Two reviewers independently screened the titles and abstracts and applied inclusion criteria: RCT design using a digital mental health intervention in a population of adults with another chronic condition, published after 2010 in French or English, and including an outcome measurement of anxiety or depression.

**Results:**

Seven hundred eight primary studies were extracted from the systematic reviews and 84 primary studies met the inclusion criteria Digital mental health interventions were significantly more effective than in-person care for both anxiety and depression outcomes. Online messaging was the most effective technology to improve anxiety and depression scores; however, all technology types were effective. Interventions partially supported by healthcare professionals were more effective than self-administered.

**Conclusions:**

While our meta-analysis identifies digital intervention’s characteristics are associated with better effectiveness, all technologies and levels of support could be used considering implementation context and population.

**Trial registration:**

The protocol for this review is registered in the National Collaborating Centre for Methods and Tools (NCCMT) COVID-19 Rapid Evidence Service (ID 75).

**Supplementary Information:**

The online version contains supplementary material available at 10.1186/s13643-023-02241-1.

## Background

Chronic diseases are the main burden on health systems in developed countries and account for almost 70% of deaths worldwide [1]. The majority of people with a chronic disease (e.g., diabetes, hypertension, chronic obstructive pulmonary disease) have more than one concurrent condition and are also at higher risk for developing comorbidities in mental health, including anxiety and depression [2]. The prevalence of depression or anxiety together with another chronic health disease is approximated to 27% in outpatients populations [3, 4]. Studies have also reported a higher prevalence of anxiety disorders in relation to different chronic diseases [5]. Increased use of health services is also observed in people living with a chronic disease and with a concomitant depressive disorder [6]. Canadians primary healthcare interdisciplinary teams, surveyed during the COVID-19 pandemic, reported a need for broadening services offering to answer the increase in encounters for mental health issues [7]. There is an urgent need for more relevant and accurate data on digital interventions in this area to prepare for an increase demand for mental health services.

Practice-based interventions in primary care settings have been shown effective to improve the management of depression in people with chronic diseases [4]. In addition, a large number of interventions using digital technologies have been evaluated for the management of depression or anxiety [8, 9], and systematic reviews indicate that they are effective in providing timely and delocalized care for college students [10]. However, it is still unclear what elements or characteristics of digital interventions for mental health are effective [10].

A rapid review provides knowledge users with data that can be readily used to inform healthcare decisions [11]. When the topic is broad, it can lead to data that lack precision to fulfil knowledge users’ needs regarding the most effective content and implementation methods. In an effort to gather data on the effectiveness of digital mental health interventions for people with a chronic disease, a rapid review of systematic reviews was completed [12] and offered only an overview of the problem. The aim of this study was to conduct a meta-analysis of the digital mental health interventions for people with comorbid physical and mental chronic diseases to compare the effect of technology systems and level of support.

## Methods

We engaged with a panel of knowledge users (clinicians, decision makers), lived experience experts (patients), review methodologists, and researchers throughout the review process, including research question development, literature screening, data interpretation and writing of results, and dissemination of findings. The panel was engaged in weekly online meeting to gather comments, present results or participate in problem solving.

### Preliminary research

This secondary analysis is based on the data from a rapid review of systematic reviews [12]. We followed guidelines outlined by the Cochrane Handbook chapter regarding Overview of Reviews and the Cochrane Rapid Reviews Methods Group [13, 14]. The review identified a large body of evidence (35 systematic reviews) showing that digital mental health interventions were effective and safe for people with chronic diseases and cancer but that the evidence was still lacking for children and youth populations. To inform the knowledge users at each step, the first stage and lessons learned while developing the project were published elsewhere [12, 15]. The lessons learned paper was published in the native language of the knowledge users (French) and described the process of engaging with our panel and how it informed the review method.

**Fig. 1:**
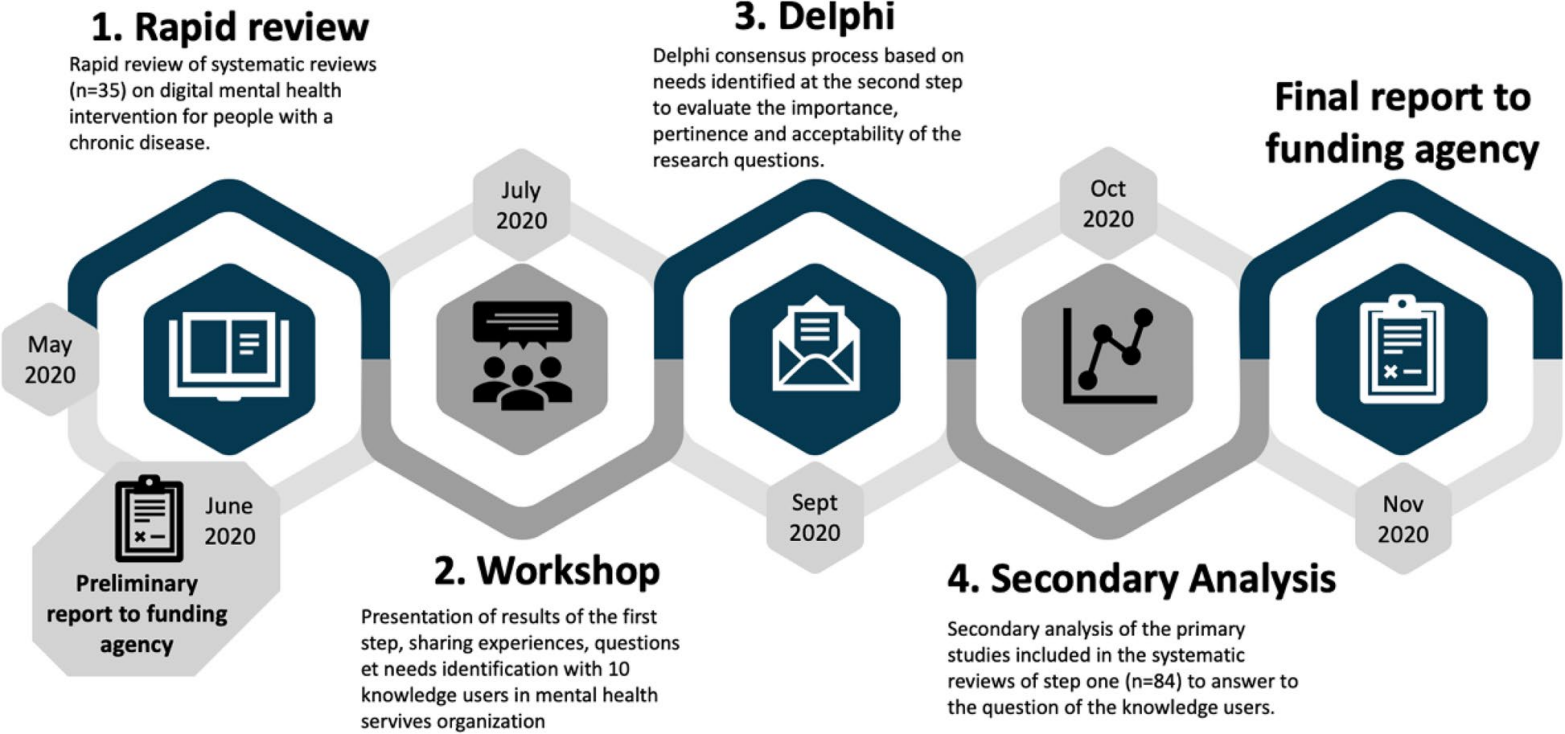
Summary of the activities and timeline

Three research activities followed the rapid review: a virtual workshop with knowledge users to present our preliminary results and gather their suggestions in nine possible research questions, a modified Delphi study to prioritize the proposed suggestions for the next stage of the review, and a secondary analysis of the primary studies identified through the rapid review. All activities of the study are summarized in Fig. 1.

### Workshop

A total of 10 knowledge users, from provincial (Quebec Ministry of Health and Social Services, Institut national d’excellence en santé et services sociaux), regional (Integrated Health and Social Services Centers) and local organizations were invited to take part in a virtual meeting on July 16th 2020 where the preliminary results from the rapid review were presented and discussed. During the workshop, knowledge users were invited to share their experience regarding digital health interventions for mental health issues, in particular in the context of the COVID-19 pandemic. They were welcome to ask questions and to comment on the findings of the rapid review and its relevance for their practice. They were also encouraged to identify knowledge gaps that could be addressed in the next stage of the review.

### Delphi study

Following the workshop with knowledge users, a summary of the main knowledge gaps identified was performed by the research team. We translated these knowledge gaps into nine review questions that were used as the basis for a modified Delphi study. We developed a questionnaire using the REDCap system [16] and sent a personalized invitation to participants in the workshop, inviting them to complete the survey. These knowledge users were also invited to suggest names of potential additional knowledge users who could have an interest in the topic. The questionnaire comprised two sections. First, participants had to rate on a 5-point Likert scale the importance, relevance, and applicability in the context of COVID-19, each of the nine potential review questions. Second, participants were invited to rank each of the nine questions (1 = most important; 9 = less important) according to their preference (Additional file 1). A total of 16 knowledge users were invited to take part in a two-round Delphi process to identify the key question for the next stage of the knowledge synthesis. All the knowledge users completed the two rounds and all questions reached consensus using median and IQR. The prioritized question after the second round was “What types of digital health interventions (according to a recognized categorization) are the most effective for the management of concomitant mental health and chronic disease conditions in adults?”

### Secondary analysis

To provide an answer to knowledge users, we conducted a secondary analysis of the primary studies included in the reviews identified in our previous rapid review, published elsewhere [1]. Based on the input from knowledge users, we added inclusion criteria at this stage. We included randomized-controlled trials (RCT) including a digital health intervention for the management of concomitant mental health and chronic disease conditions in adults, published during the last 10 years (since 2010) in French or English, and including an outcome measurement of anxiety and/or depression.

All the primary studies referenced in the 35 systematic reviews were extracted and included for the citation screening. Six reviewers individually performed screenings for titles, abstracts and then full text using pilot-tested standardized forms on 25 citations for the first level of screening. All citations were reviewed by two reviewers independently at the first level of screening. A standardized extraction form was developed that included study characteristics (e.g., authors, country, design), intervention characteristics (e.g., type of digital intervention), and outcomes reported (measurement tools used, means, standard deviation, and time of measurement). Data were extracted by four research associates, and a senior investigator (MPG) completed a quality appraisal of all extracted data. Information related to the study characteristics (first author, date of publication, country, population, health condition, type of intervention, outcomes measured, means, standard deviations, author’s conclusions) were extracted directly in the DistillerSR tool [17].

As knowledge users were interested in obtaining evidence on specific types of digital health interventions for concomitant mental health and chronic health conditions, we looked for existing classifications of digital health tools to sort interventions. We consulted the WHO *Classification of digital health interventions* [18] and previous reviews on digital mental health solutions [19, 20]. Given the limitations of existing categorizations, we used our own system which considered the technology system, the synchronous or asynchronous nature of the intervention, and the level of professional support (self-administration, partially guided, guided). The classification used ten technology systems categories (not exclusive): Internet or website, computer software, mobile application, electronic messaging (email, SMS), electronic health record, telehealth (telemedicine, telepsychiatry), virtual reality/augmented reality, robot, connected devices, and other system.

We completed a meta-analysis of the standardized means difference (SMD) with an analysis of heterogeneity (χ^2^and *I* ^2^) for the two outcomes of interest. We used Cohen’s *D*, fixed-effects meta-analysis and the R software for data analysis.

The Cochrane Risk of Bias for Intervention Studies (ROBIS) tool was completed by two investigators (MS, MPG) to assess the probability of bias in the included studies. Five types of biases were considered: (1) risk of bias arising from the randomization process; (2) risk of bias due to deviations from the intended interventions; (3) missing outcome data; (4) risk of bias in the measurement of the outcomes; and (5) risk of bias in the detection of the reported results. An overall risk of bias was also assessed.

We report our results based on the Preferred Reporting Items for Systematic Reviews and Meta-Analyses (PRISMA) Statement [21].

## Result

**Fig. 2:**
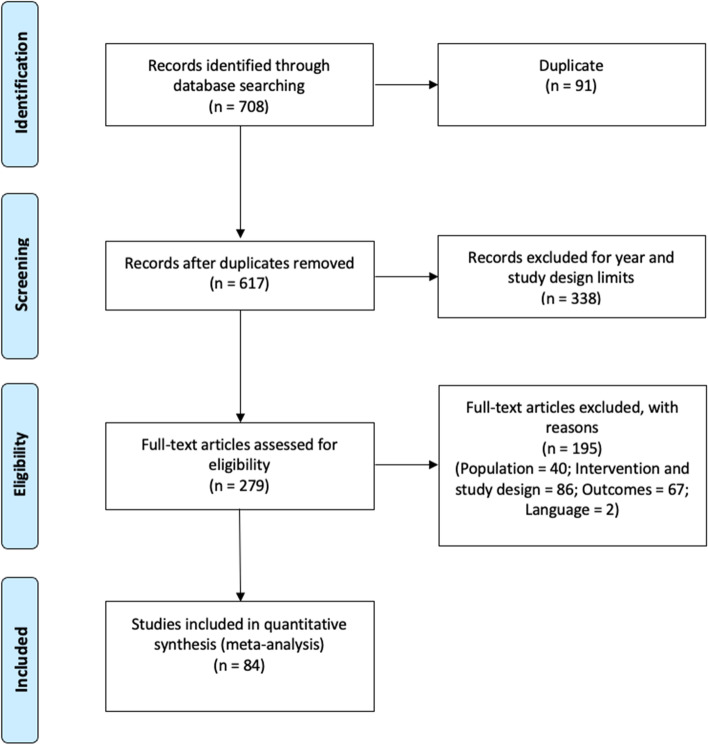
Study flow diagram: meta-analysis. Moher D, Liberati A, Tetzlaff J, Altman DG, The PRISMA Group (2009). Preferred Reporting Items for Systematic Reviews and Meta-Analyses: The PRISMA Statement. PLoS Med 6(6): e1000097. doi:http://orcid.org/10.1371/journal.pmed1000097

The flow diagram of studies included in the secondary analyses is presented in Fig. 2. All individual primary studies included in the systematic reviews of the rapid review were considered.

A total of 708 primary studies were identified from the systematic reviews included in the rapid review. A total of 429 primary studies were excluded at title and abstract screening stage (duplicates 91; publication year or study design 338). Following screening of full text, we excluded 195 records with reasons (population 40; intervention and study design 86; outcomes 67; language 2), resulting in a total of 84 primary studies included in the secondary meta-analysis.

### Characteristics of included studies

We included primary studies published between 2010 and 2019. Those studies comprised a total sample of 11,037 participants. Countries where the studies were conducted included Sweden (23), the USA (18), Australia (14), Netherlands (9), United Kingdom (7), Germany (6), Switzerland (2), Norway (1), Canada (1), Jordan (1), New Zealand (1), and South Korea (1).

Most studies described interventions performed in the community (33%) and targeted a mixed gender adult population (91%). All studies evaluated digital interventions to manage and treat mental health issues, and a majority (80%) were based on cognitive behavioural therapy (CBT). Most studies compared digital health interventions to usual care (76%), although some studies compared two or more digital interventions (24%).

The complete description of included studies is presented in Table 1 and interventions are described under the framework of digital mental health interventions including a description of the system use, the function of the digital intervention, the time, and the level of facilitation (Additional files 2 and 3).

**Table 1:** Description of included studies in the meta-analysis

**Author, year**	**Country**	**Chronic disease**	**Sample size**	**Type of digital technology intervention**	**Outcome measure**
Aguado [22] 2012	USA	Cancer	199	DVD/video	Depression/anxiety
Andersson [23] 2012	Sweden	Obsessive-compulsive disorder	101	Internet or website	Depression
Andersson [24] 2012	Sweden	Generalized anxiety disorder	81	Internet or website/mobile application	Anxiety/depression
Andersson [25] 2012	Sweden	Generalized anxiety disorder	204	Internet or website/email (SMS)	Anxiety/depression
Bani [26] 2019	Jordan	Breast cancer	80	Virtual reality	Anxiety
Bell [27] 2012	New Zealand	Generalized anxiety disorder	83	Internet or website	Anxiety
Berger [28] 2011	Switzerland	Major depressive disorder	761	Internet or website	Depression
Bergström [29] 2010	Sweden	Panic disorder	113	Internet or website/email (SMS)	Depression
Boele [30] 2018	Netherlands	Cancer	115	Internet or website	Depression
Bond [31] 2010	USA	Diabetes	62	Internet or website	Depression
Bowler [32] 2012	United Kingdom	Major depressive disorder and generalized anxiety disorder	63	Computer (program or software)	Depression/anxiety
Braamse [33] 2016	Netherlands	Transplantation for hematological malignancies	95	Internet or website	Depression/anxiety
Bromberg [34] 2012	USA	Chronic migraine	189	Internet or website	Anxiety
Buhrman [35] 2013a	Sweden	Chronic pain	72	Internet or website	Depression/anxiety
Buhrman [36] 2011	Sweden	Chronic pain	60	Internet or website	Depression/anxiety
Buhrman [37] 2013b	Sweden	Chronic pain	76	Internet or website	Depression/anxiety
Buhrman [38] 2015	Sweden	Chronic pain and major depressive disorder and generalized anxiety disorder	52	Internet or website	Depression/anxiety
Carlbring [39] 2011	Sweden	Social phobia and generalized anxiety disorders	54	Internet or website	Depression/anxiety
Carrard [40] 2011	Switzerland	Eating disorder	74	Internet or website	Depression
Cohn [41] 2014	USA	Diabetes	49	Internet or website	Depression/anxiety
Cooper [42] 2011	United Kingdom	Sclerosis	24	Computer (program or software)	Depression
Damholdt [43] 2016	Germany	Breast cancer	157	Internet or website/email (SMS)	Depression/anxiety
Dear [44] 2015	Australia	Chronic pain	490	Internet or website/email (SMS)	Depression/anxiety
Dear [45] 2013	Australia	Chronic pain	63	Internet or website/email (SMS)	Depression/anxiety
Devi [46] 2014	United Kingdom	Angina	94	Internet or website/email (SMS)	Depression/anxiety
Drozd [47] 2014	Norway	HIV	67	Internet or website/email (SMS)	Depression
Engel [48] 2015	USA	Post-traumatic stress disorder	80	Internet or website	PTSD
Everitt [49] 2013	United Kingdom	Irritable bowel syndrome	135	Internet or website	Anxiety
Farrer [50] 2011	Germany	Major depressive disorder	155	Internet or website	Depression
Friesen [51] 2017	Canada	Fibromyalgia	60	Internet or website/email (SMS)	Depression/anxiety
Glozier [52] 2013	Australia	Cardiovascular disease	562	Internet or website	Depression/anxiety
Hedborg [53] 2011	Sweden	Migraine	76	Internet or website/email (SMS)	Depression
Hedman [54] 2014	Sweden	Generalized anxiety disorder	81	Internet or website	Anxiety
Hedman [55] 2011	Sweden	Hypochondriasis anxiety	81	Internet or website/telehealth	Depression/anxiety
Hesser [56] 2012	Sweden	Chronic tinnitus	99	Internet or website	Depression/anxiety
Ivarsson [57] 2014	Sweden	Post-traumatic stress disorder	62	Internet or website	Depression/anxiety
Jacobi [58] 2012	Germany	Eating disorder	126	Internet or website/email (SMS)	Depression
Jasper [59] 2014	Germany	Tinnitus	128	Internet or website/email (SMS)	Depression/anxiety
Johansson [60] 2015	Sweden	Traumatic brain injury or stroke	34	Internet or website/telehealth	Depression/anxiety
Johnston [61] 2011	Australia	Generalized anxiety disorder	139	Internet or website/email (SMS)	Anxiety/depression
Knaevelsrud [62] 2015	Germany	Post-traumatic stress disorder	159	Internet or website/telehealth/email (SMS)	Depression/anxiety
Kok [63] 2014	Netherlands	Phobia	212	Internet or website	Depression/anxiety
Kraaijt [64] 2010	Netherlands	HIV	73	Internet or website/computer (program or software)/CD-ROM	Depression
Kuhn [65] 2017	USA	Post-traumatic stress disorder	120	Mobile application	Depression
Lewis [66] 2017	United Kingdom	Post-traumatic stress disorder	42	Internet or website/email (SMS)	Depression/anxiety
Littleton [67] 2016	USA	Post-traumatic stress disorder	87	Internet or website	Depression/anxiety
Ljótsson [68] 2011	Sweden	Irritable bowel syndrome	195	Internet or website/email (SMS)	Anxiety/depression
Ljótsson [69] 2010	Sweden	Irritable bowel syndrome	85	Internet or website	Depression
Lundgren [70] 2016	Sweden	Major depressive disorder and heart failure	50	Internet or website/email (SMS)	Depression/anxiety
Mailey [71] 2010	USA	Mental health disorder	51	Computer (program or software)	Anxiety/depression
Migliorini [72] 2016	Australia	Spinal cord injury	59	Internet or website	Depression/anxiety
Newby [73] 2013	Australia	Major depressive disorder (MDD) and generalized anxiety disorder	109	Internet or website	Depression/anxiety
Newby [74] 2014	Australia	Major depressive disorder and generalized anxiety disorder	109	Internet or website/email (SMS)	Depression/anxiety
Newby [75] 2017	Australia	Diabetes	106	Internet or website/email (SMS)	Depression/anxiety
Nordgren [76] 2014	Sweden	Generalized anxiety disorder	100	Internet or website/email (SMS)	Anxiety/depression
Paxling [77] 2011	Sweden	Generalized anxiety disorder	89	Internet or website	Anxiety/depression
Peters [78] 2017	Sweden	Chronic pain	284	Internet or website	Depression/anxiety
Possemato [79] 2015	USA	Post-traumatic stress disorder	20	Internet or website	Depression
Robinson [80] 2010	Australia	Generalized anxiety disorder	150	Internet or website/email (SMS)	Anxiety/depression
Rosmarin [81] 2010	USA	Generalized anxiety disorder	125	Internet or website	Depression
Roy [82] 2010	USA	Generalized anxiety disorder	1004	Internet or website	Depression/anxiety
Ruehlman [83] 2012	USA	Chronic pain	305	Internet or website	Depression/anxiety
Ruwaard [84] 2010	Netherlands	Panic disorder	58	Internet or website	Depression/anxiety
Sanchez [85] 2011	United Kingdom	Bulimia	76	Internet or website/email (SMS)	Depression/anxiety
Seekles [86] 2011	Netherlands	Major depressive disorder	120	Internet or website/telehealth	Depression/anxiety
Sexton [87] 2010	USA	Infertility	43	Internet or website	Depression/anxiety
Shigaki [88] 2013	USA	Arthritis	108	Internet or website	Depression
Silfvernagel [89] 2012	Sweden	Panic disorder	57	Internet or website/email (SMS)	Depression/anxiety
Spence [90] 2014	Australia	Post-traumatic stress disorder	125	Internet or website/email (SMS)	Depression/anxiety
Spence [91] 2011	Australia	Post-traumatic stress disorder	44	Internet or website	Depression/anxiety
Titov [92] 2010	Australia	Major depressive disorder	141	Internet or website/email (SMS)	Depression/distress
Titov [93] 2010	Australia	Generalized anxiety disorder	86	Internet or website/email (SMS)	Depression/anxiety
Trompetter [94] 2014	Netherlands	Chronic pain	238	Internet or website	Depression/anxiety
Trudeau [95] 2015	USA	Chronic pain	228	Internet or website	Depression/anxiety
van Ballegooijen [96] 2013	Netherlands	Panic disorder	126	Internet or website	Depression/anxiety
Varley [97] 2011	United Kingdom	Generalized anxiety disorder	262	Internet or website	Depression/anxiety
Vernmark [98] 2010	Sweden	Major depressive disorder	85	Internet or website/email (SMS)	Depression/anxiety
Weise [99] 2016	Germany	Tinnitus	124	Internet or website	Depression/anxiety
Willems [100] 2017	Netherlands	Cancer	518	Internet or website	Depression
Williams [101] 2010	USA	Fibromyalgia	118	Internet or website	Depression/anxiety
Wilson [102] 2015	USA	Chronic pain	114	Internet or website	Depression
Wilson [103] 2017	USA	Chronic disease	47	Internet or website	Depression/distress
Wims [104] 2010	Australia	Panic disorder	59	Internet or website/email (SMS)	Depression
Wootton [105] 2013	Australia	Obsessive-compulsive disorder	56	Internet or website	Depression/anxiety
Yun [106] 2012	South Korea	Cancer	273	Internet or website	Depression/anxiety

A summary of the estimated effect size for each intervention characteristics, heterogeneity, and inconsistency for all comparisons, and for both outcomes are presented in Table 2.

**Table 2:** Summary of comparative intervention characteristics

Interventions characteristics	*n* participants	Effect estimate (95% CI)	Heterogeneity, *χ*² (*p* value)	Inconsistency, *I* ^2^ (%)
*Anxiety outcomes*				
Mobile applications	76	− 0.52 (− 0.04, − 1.00)	N/A	N/A
Digital video disc (other)	220	− 0.15 (0.11, − 0.42)	N/A	N/A
Computer software	114	− 0.57 (− 0.18, − 0.96)	3.08 (0.08)	67.55
Connected devices	51	− 0.22 (0.33, − 0.77)	N/A	N/A
Electronic messaging	2700	− 0.48 (− 0.39, − 0.56)	95.30 (< .0005)	79.01
Internet/website	8305	− 0.39 (− 0.35, − 0.44)	396.02 (< .0005)	85.61
Telehealth/telemedecine	394	− 0.50 (− 0.29, − 0.70)	21.48 (< .0005)	86.03
Virtual reality	80	− 1.73 (− 1.21, − 2.25)	N/A	N/A
Self− administred	5312	− 0.35 (− 0.30, − 0.41)	231.19 (< .0005)	87.46
Partially guided	3206	− 0.46 (− 0.39, − 0.53)	155.93 (< .0005)	82.04
Guided	201	− 0.46 (− 0.16, − 0.76)	36.55 (< .0005)	94.53
Overall effect	8719	− 0.40 (− 0.35, − 0.44)	428.75 (< .0005)	85.77
*Depression outcomes*				
Mobile applications	196	− 0.26 (0,02, − 0.55)	0.09 (0.768)	0
Digital video disc (other)	273	− 0.10 (0.14, − 0.34)	1.41 (0.241)	29.01
Computer software	191	− 0.55 (− 0.26, − 0.85)	5.71 (0.134)	47.42
Connected devices	51	− 0.13 (0.42, − 0.68)	N/A	N/A
Electronic messaging	2915	− 0.48 (− 0.40, − 0.56)	146.62 (< .0005)	83.63
Internet/website	9492	− 0.33 (− 0.29, − 0.37)	299.91 (< .0005)	76.99
Telehealth/telemedecine	394	− 0.75 (− 0.54, − 0.96)	15.18 (0.002)	80.24
Self-administred	6379	− 0.28 (− 0.23, − 0.33)	113.53 (< .0005)	65.65
Partially guided	3470	− 0.43 (− 0.36, − 0.50)	176.59 (< .0005)	81.88
Guided	121	0.16 (0.53, − 0.22)	4.96 (0.026)	79.82
Depression overall effect	9970	− 0.33 (− 0.29, − 0.37)	312.90 (< .0005)	76.35

*CI* Confidence interval

### Global mean differences

The first analysis aimed at the global group differences of any digital intervention compared to usual care or another digital intervention to manage anxiety or depression for people with any concomitant chronic condition.

**Fig. 3:**
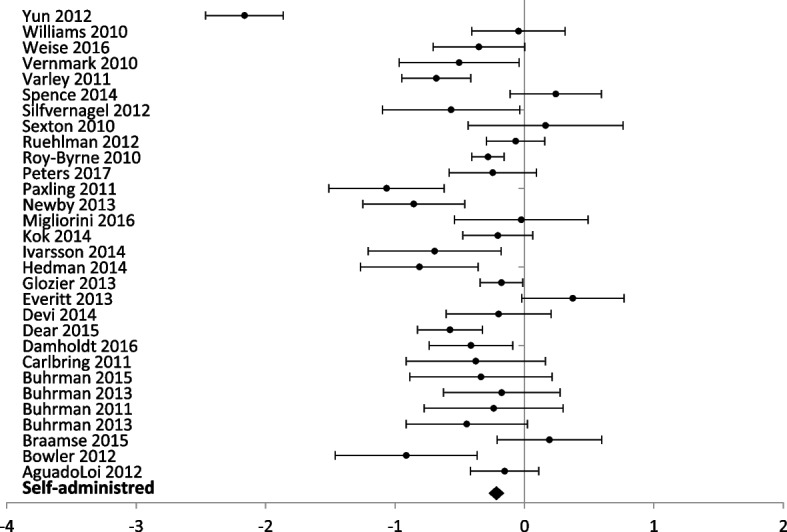
Forest plot: subgroup analysis of self-directed interventions for any digital intervention vs. usual care or another digital intervention to manage anxiety in people with any concomitant chronic condition

A total of 62 studies including 8719 participants with anxiety outcomes measures were included in the meta-analysis of the overall effect of digital interventions on anxiety outcomes (Additional file 4). The results showed a significant decrease in the anxiety score related to digital health interventions compared to usual care or another digital intervention [standardized mean difference (SMD) = − 0.40; 95% confidence interval (CI) = − 0.35; − 0.44)] (Fig. 3). Although heterogeneity is high between studies (*I*^2^ = 85.77%) the results are consistent across studies (Table 2).

**Fig. 4:**
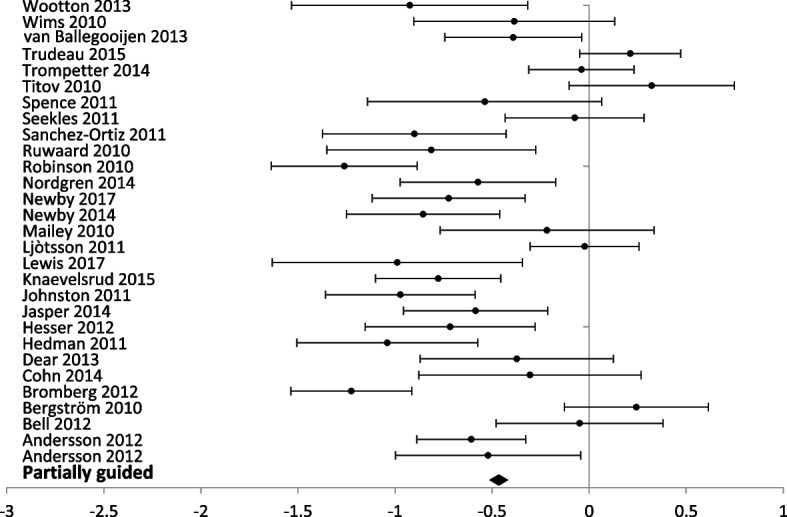
Forest plot: subgroup analysis of partially guided interventions for any digital intervention vs. usual care or another digital intervention to manage anxiety in people with any concomitant chronic condition

Regarding depression outcomes, we conducted a meta-analysis including 75 studies with 9970 participants. Figure 4 shows a significant reduction in depression scores associated with the use of digital health interventions compared to usual care (SDM = − 0.33; 95% CI [− 0.29, − 0.37]). Heterogeneity is also high for this overall comparison (*I*^2^ = 76.35%) with results consistent across studies (Table 2).

### Level of professional support

**Fig. 5:**
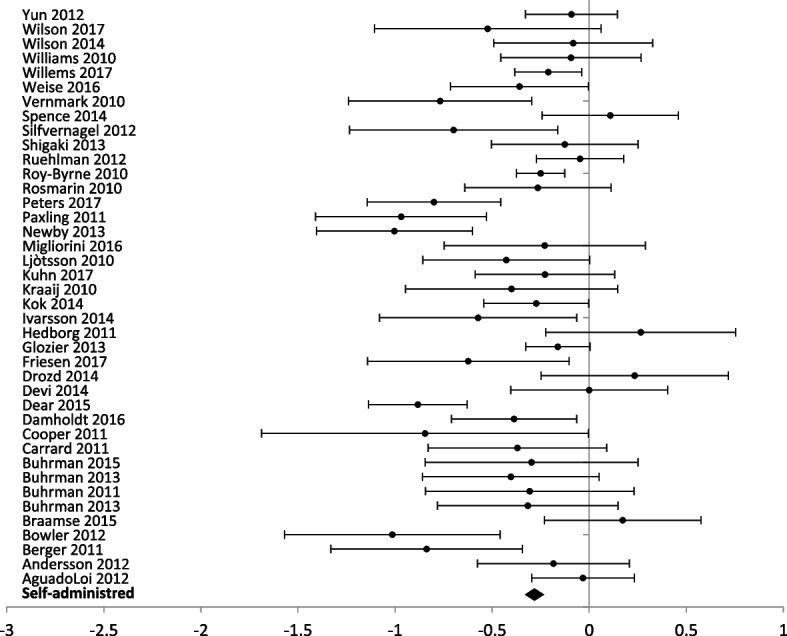
Forest plot: subgroup analysis of self-directed interventions for any digital intervention vs. usual care or another digital intervention to manage depression in people with any concomitant chronic condition

**Fig. 6:**
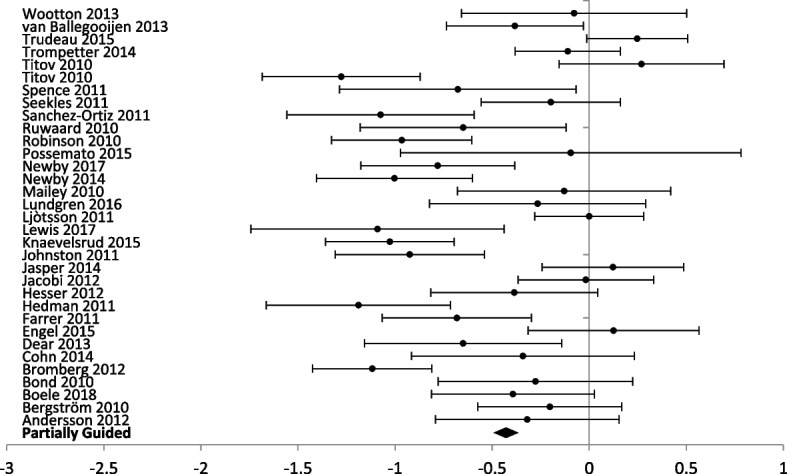
Forest plot: subgroup analysis of partially guided interventions for any digital intervention vs. usual care or another digital intervention to manage depression in people with any concomitant chronic condition

The forest plots for comparing the level of professional support are presented for anxiety (Figs. 3 and 4) and depression (Figs. 5 and 6) outcomes separately.

Self-administration was the delivery method in 30 studies (5312 participants) with anxiety outcomes and 40 studies (6379 participants) reporting depression outcomes. Self-administered delivery showed a significant decrease in anxiety scores [SMD − 0.35 (− 0.30, − 0.41)] and depression scores [(SMD − 0.28 (− 0.23, − 0.33)].

Partial support by a healthcare provider was used in 29 studies (3206 participants) reporting anxiety outcomes, and 33 studies (3470 participants) reporting depression outcomes. Partial support showed a significant decrease in anxiety [SMD – 0.46 (− 0.39, − 0.53)] and depression scores [SMD – 0.43 (− 0.36, − 0.50)].

Interventions entirely guided by a healthcare professional were used in three studies (201 participants) reporting anxiety outcomes and two studies (121 participants) reporting depression outcomes. Interventions entirely supported by healthcare professionals showed a significant difference for anxiety scores [SMD − 0.46 (− 0.16, − 0.76)] but no significant difference between groups for depression scores [SMD 0.15 (0.53, − 0.21)].

### Type of technology

**Fig. 7:**
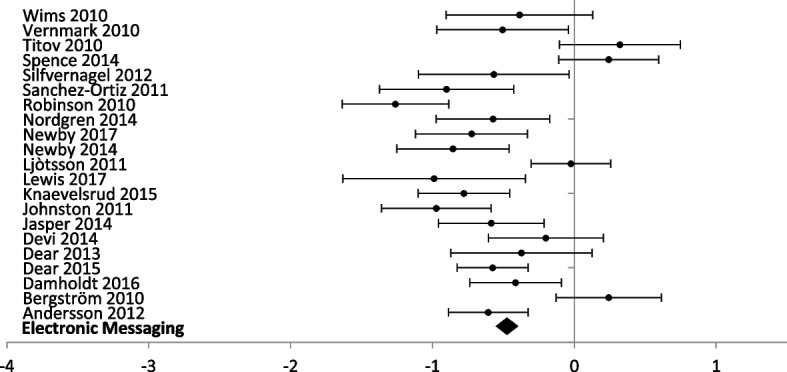
Forest plot: subgroup analysis of electronic messaging interventions for any digital intervention vs. usual care or another digital intervention to manage anxiety in people with any concomitant chronic condition

**Fig. 8:**
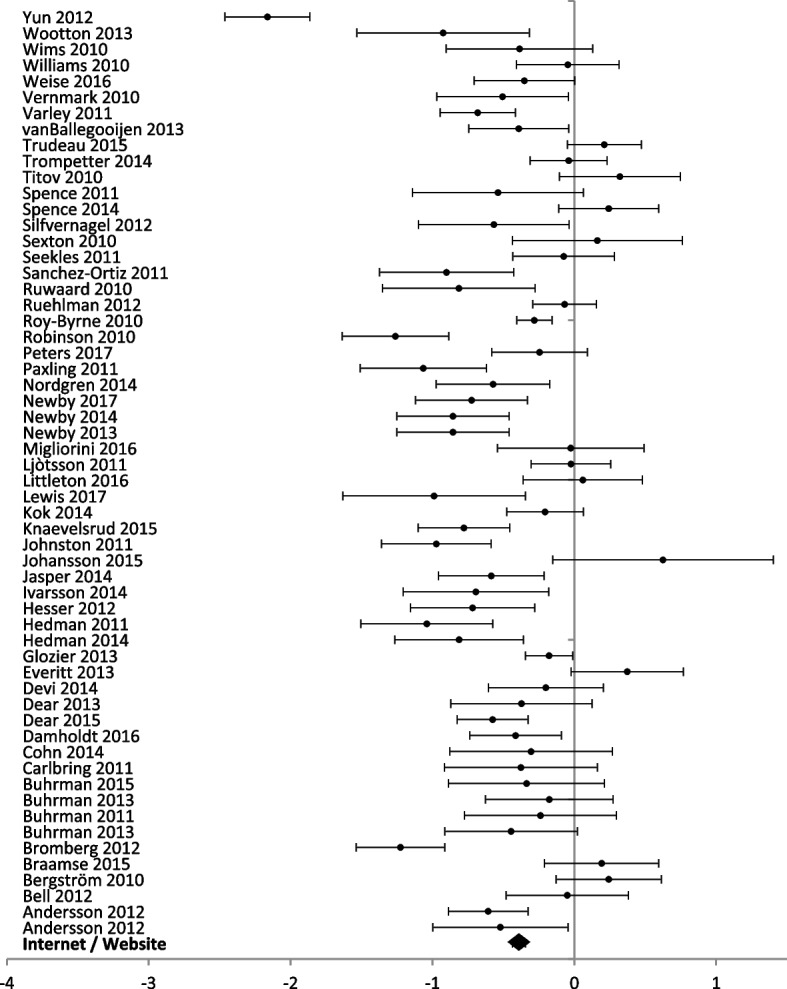
Forest plot: subgroup analysis of internet or website interventions for any digital intervention vs. usual care or another digital intervention to manage anxiety in people with any concomitant chronic condition

**Fig. 9:**
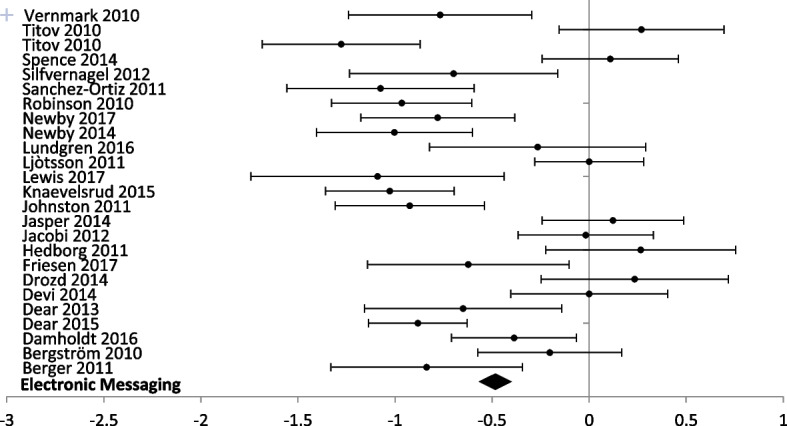
Forest plot: subgroup analysis on electronic messaging interventions for any digital intervention vs. usual care or another digital intervention to manage depression in people with any concomitant chronic condition

**Fig. 10:**
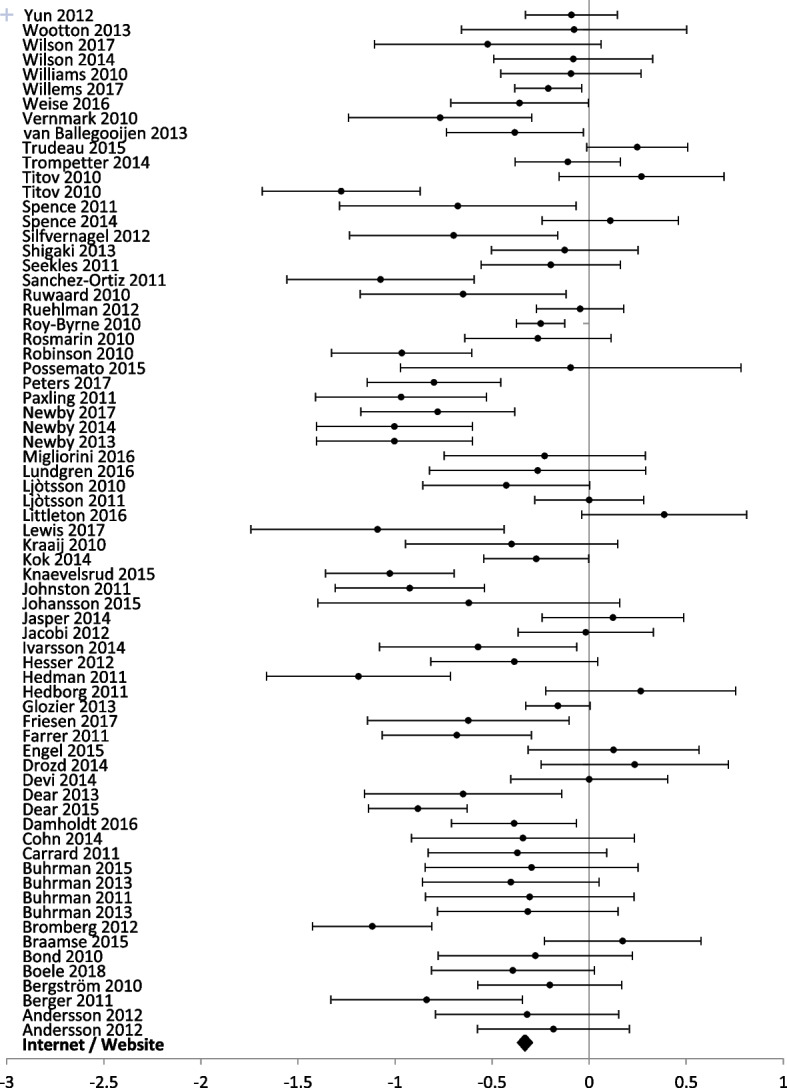
Forest plot: subgroup analysis on internet and website interventions for any digital intervention vs. usual care or another digital intervention to manage depression in people with any concomitant chronic condition

Eight different technologies were used in studies reporting anxiety outcomes and half (*n* = 4) had multiple reporting to perform a meta-analysis (Figs. 7 and 8). For studies reporting a depression outcome, seven technologies were used, and five of them had more than one study (Figs. 9 and 10). Forest plots that include all studies are presented in Additional files 6 and 7.

Electronic messaging was used in 23 studies (2700 participants) reporting anxiety outcomes, and 25 studies (2915 participants) reporting depression outcomes. Electronic messaging showed significant improvement in anxiety scores [SMD − 0.48 (− 0.39, − 0.56)] and depression scores [SMD − 0.48 (− 0.40, − 0.56)].

Internet and website technologies were used in 58 studies (8305 participants) reporting anxiety outcomes, and 70 studies (9492 participants) reporting depression outcomes. Internet and website interventions showed significant improvement in anxiety [SMD − 0.39 (− 0.35, − 0.44)] and depression scores [SMD − 0.33 (− 0.29, − 0.37)].

Telehealth and telemedicine were used in four studies (394 participants) reporting anxiety and/or depression outcomes. Telehealth and telemedicine showed significant improvement in anxiety [SMD − 0.50 (− 0.29, − 0.70)] and depression scores [SMD − 0.75 (− 0.54, − 0.96)].

A computer software was used in two studies (114 participants) reporting anxiety outcomes and four studies (191 participants) with depression outcomes. Computer software showed significant improvement in anxiety [SMD − 0.57 (− 0.18, − 0.96)] and depression scores [SMD − 0.55 (− 0.26, − 0.85)].

Mobile applications were used in one study (76 participants) with anxiety outcomes and two studies (196 participants) with depression outcomes. Mobile applications showed significant improvement for anxiety scores [SMD − 0.52 (− 0.04, − 1.00)], but no significant difference between groups for depression scores [SMD − 0.26 (0.02, − 0.55)].

Connected devices were used in one study with 51 participants and showed no differences between groups for anxiety [SMD − 0.22 (0.33, − 0.77)] and depression scores [SMD − 0.13 (0.42, − 0.68)]

Virtual reality was used in one study with 80 participants with anxiety outcomes and showed significant improvement on anxiety scores [SMD − 1.73 (− 1.21, − 2.25)].

One other type of technology in the form of Digital Video Disk (DVD) was used in one study (220 participants) with anxiety outcomes and two studies (273 participants) with depression outcomes. DVD interventions showed no differences between groups for anxiety [SMD − 0.15 (0.11, − 0.42)] and depression scores [SMD − 0.10 (0.14, − 0.34)].

### Risk of bias

**Fig. 11:**
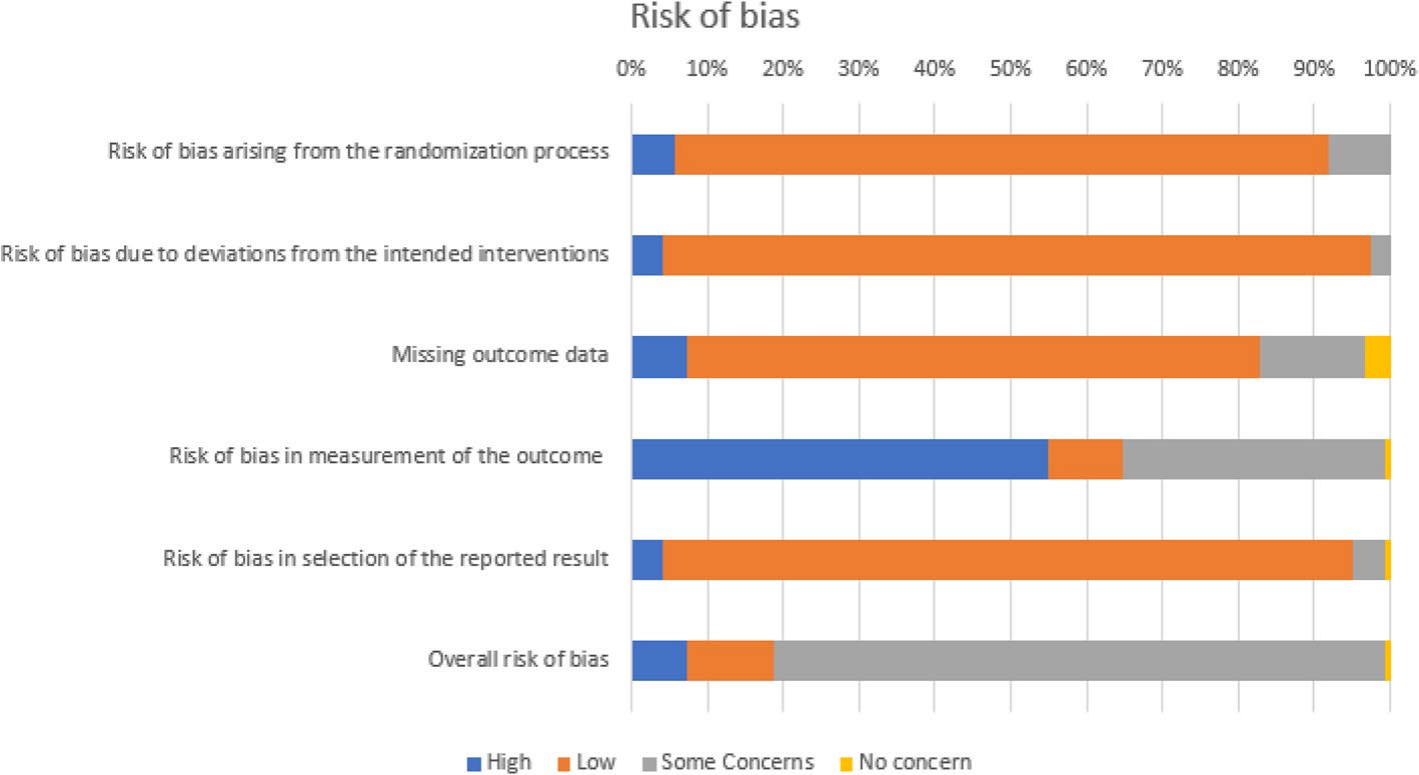
Assessment of the risk of bias across studies

Figure 11 presents the risk of bias across studies for each domain. Most of the included studies showed an overall low risk of bias. However, risk of bias was generally high for the Domain 4: *Risk of bias in the measurement of the outcomes*. In fact, blinding of study participants was not done in most studies and outcomes were self-reported, leading to a high risk of performance bias. This bias is present across studies because it is not possible in a behavioral intervention and would eventually lead to an overestimation of the effect.

## Discussion

This knowledge synthesis aimed to rapidly provide evidence for knowledge users regarding the types of digital mental health interventions that were the most effective for people living with a concomitant chronic disease. This secondary analysis answers a specific research question based on knowledge users’ needs and prioritization. Thus, preliminary work in the form of a workshop and a two-round Delphi study were conducted to identify the top-priority question for knowledge users. This question was “What types of digital health interventions are the most effective for the management of concomitant mental health and chronic disease conditions in adults?”. A total of 84 primary studies including anxiety and depression outcomes were identified from the systematic reviews.

Overall, the results show that digital health interventions are effective to manage mental health issues in adults living with a concomitant chronic condition. The magnitude of the effect varies for anxiety and depression, and heterogeneity is generally high, but the effect size and direction are consistent across studies. Subgroup analyses show that digital mental health interventions with partial support from a healthcare provider have a larger effect size than self-administered interventions. These results are in line with what is known for other populations [107, 108]. There is not enough evidence to conclude in the effectiveness of digital interventions that are completely guided by a healthcare professional because of the lack of studies in this category. One key challenge of self-administered digital mental health interventions is sustaining engagement and reducing dropout [109, 110]. Partially supported interventions are mitigating these challenges by improving interactivity and personalization [111, 112]. However, it is also documented that patients could prefer to only interact with a platform instead than talking with a healthcare professional, highlighting a need for flexible interventions [113]. This meta-analysis shows that partially supported and self-directed digital mental health interventions can be used for patients with chronic diseases and could save clinical time and resources as well as care engagement.

Regarding the type of technology used, our analyses show that the most effective type of intervention is electronic messaging, but that all types of technologies are effective for both anxiety and depression scores. This finding adds to the literature on the use of digital mental health to reduce disparities, while considering that technology have varying value across population (e.g., elder, lower socio-economic). Indeed, as our results show that all types of technologies are equally or more effective than usual care, stakeholders could choose and implement interventions in relation with the needs of the population. For example, decision-makers can tailor their choices with respect to cost, ease of access or easing stigma barriers [114].

More research will be needed for newer technologies, such as mobile apps and virtual reality, which have showed effectiveness only in a small number of studies with a large confidence interval.

The significant statistical heterogeneity observed between studies in every comparison is not surprising and could be likely due to differences in comorbidity, outcome measure used, and content of the intervention. However, patterns shown in this meta-analysis are useful for clinical use and implementation.

This knowledge synthesis was informed by knowledge users in order to validate the review questions considering their needs and identify knowledge gaps that would require more evidence. We used a two-stage process, starting with a rapid review of systematic reviews followed by a secondary analysis of the primary studies. Although we used a systematic approach for selecting these studies, a major limitation is that more recent studies were not included in the analyses. To meet the requirement of the funding agency and the urgent need for evidence in the current pandemic, we considered only the most recent studies (published from 2010) from the included reviews. We also assessed the risk of bias in the included studies.

Results from the meta-analyses should be interpreted with caution since heterogeneity was generally high. Further analyses, including subgroup analyses for different populations, are needed to provide a more detailed and nuanced portrait of the effectiveness of digital mental health interventions. Furthermore, sensitivity analyses, notably by considering the risk of bias related to the lack of blinding of participants, would be required to minimize the risk of an overestimation of the effect. Some studies used multiples systems in the same intervention, which could overestimate the effect size. We also merged together comparator groups, which could affect the interpretation of results for clinical application. Finally, we cannot rule out the possibility of publication bias, as well as other factors that could lessen the level of confidence in the reported effects.

Available evidence suggests that digital health interventions such as internet-based cognitive behavioural therapy (iCBT) could be effective and provide an alternative to face-to-face psychological interventions to manage mental health issues in adults living with a concomitant chronic condition. In the context of the COVID-19 pandemic, digital technologies have played a key role in healthcare. Many of these innovations support the care of people in need of medical attention, including those with chronic illnesses. In response to the current crisis, but also to better prepare for the post-crisis and future crises, digital mental health interventions could be a useful tool to manage mental health problems in people living with chronic conditions.

## Conclusion

This knowledge synthesis provides an overview of the current evidence regarding the use of digital health interventions to improve mental health in people living with a chronic condition. Knowledge users’ most urgent need was for evidence on which type of digital interventions to use for mental health management. While our meta-analysis indicates different levels of effectiveness associated with digital interventions’ characteristics, all technologies and levels of support can be used with consideration of implementation context and population, and self-administered and partially supported interventions could help save time and resources as well as supporting engagement in care.

## Supplementary Information

**Additional file 1.** Nine research questions identified by the knowledge user group (translated from French).

**Additional file 2.** Classification framework of digital mental health interventions, reproduced from Gagnon et al. 2022.

**Additional file 3:** **Additional Table 3.** Classification of the *System:* 1. Internet or Website 2. Computer (software) 3. Mobile app 4. Electronic messaging (email, SMS) 5. Electronic health record 6. Telehealth (telemedicine, telepsychiatry) 7. Virtual reality/ augmented reality 8. Robot 9. Connected devices 10. Social media 11. Other system;*Function:* A.Decision support a)Screening b)Prompts and alerts B. Communication a. Transmission of information (one way) b. Communication (with healthcare provider) c. Communication (peer to peer, e.g., virtual peer group for clients) C. Therapy a. Cognitive Behavioural Therapy (CBT) b. Other psychotherapy c. Gamification D. Monitoring a. Provider monitoring b. Self-monitoring; *Time: *= Synchronous + Asynchronous. *Facilitation: G*. Entirely supported by healthcare providers* PG.*Partially supported by healthcare providers S. Self-administered.

**Additional file 4.** Forest plot: Subgroup analysis of self-directed interventions for any digital intervention vs. usual care or another digital intervention to manage anxiety in people with any concomitant chronic condition.

**Additional file 5.** Forest plot: Subgroup analysis on level of professional support for any digital intervention vs. usual care or another digital intervention to manage depression in people with any concomitant chronic condition.

**Additional file 6.** Forest plot: Subgroup analysis on type of technology for any digital intervention vs. usual care or another digital intervention to manage anxiety in people with any concomitant chronic condition.

**Additional file 7.** Forest plot: Subgroup analysis on type of technology for any digital intervention vs. usual care or another digital intervention to manage depression in people with any concomitant chronic condition.

## Data Availability

The datasets used and/or analyzed during the current study are available from the corresponding author on reasonable request.

## Abbreviations

QIR: Interquartile range
RCT: Randomized-controlled trials
WHO: World Health Organization
SMD: Standardized means difference
ROBIS: Risk of Bias for Intervention Studies
PRISMA: Preferred Reporting Items for Systematic Reviews and Meta-Analyses
CBT: Cognitive behavioural therapy
CI: Confidence interval
iCBT: Internet-based cognitive behavioural therapy
