# Supplementary material for: The impact of technology systems and level of support in digital mental health interventions: a secondary meta-analysis
Source: Syst Rev. 2023 May 4;12:78. doi: 10.1186/s13643-023-02241-1 (PMC10157597; doi:10.1186/s13643-023-02241-1)
Supplement: Supplementary file 1 — Additional file 1. Nine research questions identified by the knowledge user group (translated from French). [file 13643_2023_2241_MOESM1_ESM.docx]

**Additional file 1.** Nine research questions identified by the knowledge user group (translated form french).

1. What are the most effective digital interventions to support concomitant mental health and physical health in adults?
2. What are the most effective digital interventions to support concomitant mental health and physical health in children and adolescents?
3. What are the most effective digital interventions to support concomitant mental health and physical health in elderly population?
4. What are the most effective digital interventions to identify mental health conditions in children and adolescents with chronic diseases?
5. What are the most effective digital interventions for early-stage mental health intervention for children and adolescents?
6. What are the barriers and enablers to the effective use of digital mental health intervention?
7. What are the conditions to enable long-term implementation and use of digital mental health interventions?
8. Are digital mental health interventions more effective than phone interventions for patients with comorbid mental and physical chronic diseases?
9. What are the digital tools to support healthcare professional in identifying patients at risk of developing mental health conditions?
