# Supplementary material for: The impact of technology systems and level of support in digital mental health interventions: a secondary meta-analysis
Source: Syst Rev. 2023 May 4;12:78. doi: 10.1186/s13643-023-02241-1 (PMC10157597; doi:10.1186/s13643-023-02241-1)
Supplement: Supplementary file 2 — Additional file 2. Classification framework of digital mental health interventions, reproduced from Gagnon et al. 2022. [file 13643_2023_2241_MOESM2_ESM.docx]

**Additional file 2.** Classification framework of digital mental health interventions, reproduced from Gagnon et al. 2022.

| **System** | **Function (*sub-function*)** | **Time** | **Facilitation** | |  |
| --- | --- | --- | --- | --- | --- |
| 1. Internet or Website | A.  Decision support  *a) Screening*  *b) Prompts and alerts* | =. Synchronous | | G. Entirely supported by healthcare providers | |
| 2. Computer (software) |  |  |  |  |  |
| 3. Mobile app |  |  |  |  |  |
| 4. Electronic messaging (email, SMS) | B.  Communication  *a.   Transmission of information (one way)*  *b.   Communication (with healthcare provider)*  *c.   Communication (peer to peer, e.g., virtual peer group for clients)* | +. Asynchronous | | PG. Partially supported  by healthcare providers | |
| 5. Electronic health record |  |  | S. Self-administered | |  |
| 6. Telehealth (telemedicine, telepsychiatry) |  |  |  | |  |
| 7. Virtual reality/ augmented reality | C.  Therapy  *a.  Cognitive Behavioural Therapy (CBT)*  *b.  Other psychotherapy*  *c.  Gamification* |  |  | |  |
| 8. Robot |  |  |  | |  |
| 9. Connected devices |  |  |  | |  |
| 10. Social media | D.   Monitoring  a. *Provider monitoring*  *b.    Self-monitoring* |  |  | |  |
| 11. Other system |  |  |  | |  |
|  |  |  |  | |  |
