# Supplementary material for: The impact of technology systems and level of support in digital mental health interventions: a secondary meta-analysis
Source: Syst Rev. 2023 May 4;12:78. doi: 10.1186/s13643-023-02241-1 (PMC10157597; doi:10.1186/s13643-023-02241-1)
Supplement: Supplementary file 3 — Additional file 3: Additional Table 3. Classification of the System: 1. Internet or Website 2. Computer (software) 3. Mobile app 4. Electronic messaging (email, SMS) 5. Electronic health record 6. Telehealth (telemedicine, telepsychiatry) 7. Virtual reality/ augmented reality 8. Robot 9. Connected devices 10. Social media 11. Other system;Function: A.Decision support a)Screening b)Prompts and alerts B. Communication a. Transmission of information (one way) b. Communication (with healthcare provider) c. Communication (peer to peer, e.g., virtual peer group for clients) C. Therapy a. Cognitive Behavioural Therapy (CBT) b. Other psychotherapy c. Gamification D. Monitoring a. Provider monitoring b. Self-monitoring; Time: = Synchronous + Asynchronous. Facilitation: G. Entirely supported by healthcare providers PG.Partially supported by healthcare providers S. Self-administered. [file 13643_2023_2241_MOESM3_ESM.docx]

Additional table 3. Classification of the

| Authors, year | System | | Function | | | Time | Facilitation | | | | Complete Classification | | | | | | | | |  |  |  |  |  |  |  |  |  |  |  |  |  |  |  |  |  |  |  |  |
| --- | --- | --- | --- | --- | --- | --- | --- | --- | --- | --- | --- | --- | --- | --- | --- | --- | --- | --- | --- | --- | --- | --- | --- | --- | --- | --- | --- | --- | --- | --- | --- | --- | --- | --- | --- | --- | --- | --- | --- |
| Admiraal,2017 | B)a.C)b.D)b.E) | | 1. | | | # | S | | | | B)a.C)b.D)b.E)1.#S | | | | | | | | |  |  |  |  |  |  |  |  |  |  |  |  |  |  |  |  |  |  |  |  |
| Aguado Loi,2012 | D)b. | | 10. | | | # | S | | | | D)b.10.[DVD/Video, MP3]#S | | | | | | | | |  |  |  |  |  |  |  |  |  |  |  |  |  |  |  |  |  |  |  |  |
| Andersson,2012 | C)a. | | 1. | | | * | S | | | | C)a.1.*S | | | | | | | | |  |  |  |  |  |  |  |  |  |  |  |  |  |  |  |  |  |  |  |  |
| Andersson,2012 | C)a.b. | | 1.3. | | | # | PG | | | | C)a.b.1.3.#PG | | | | | | | | |  |  |  |  |  |  |  |  |  |  |  |  |  |  |  |  |  |  |  |  |
| Andersson,2012 | A)a.B)b.c.C)a.D)a. | | 1.4. | | | *# | PG | | | | A)a.B)b.c.C)a.D)a.1.4.*#PG | | | | | | | | |  |  |  |  |  |  |  |  |  |  |  |  |  |  |  |  |  |  |  |  |
| Andrews,2011 | C)a. | | 2.4. | | | # | PG | | | | C)a.2.4.#PG | | | | | | | | |  |  |  |  |  |  |  |  |  |  |  |  |  |  |  |  |  |  |  |  |
| B Mohammad,2019 | C)c. | | 7. | | | * | G | | | | C)c.7.*G | | | | | | | | |  |  |  |  |  |  |  |  |  |  |  |  |  |  |  |  |  |  |  |  |
| Beatty,2016 | C)a.D)b. | | 1.4. | | | # | S | | | | C)a.D)b.1.4.#S | | | | | | | | |  |  |  |  |  |  |  |  |  |  |  |  |  |  |  |  |  |  |  |  |
| Bell,2012 | C)a.D)b. | | 1. | | | *# | PG | | | | C)a.D)b.1.*#PG | | | | | | | | |  |  |  |  |  |  |  |  |  |  |  |  |  |  |  |  |  |  |  |  |
| Berger,2011 | B)b.C)a. | | 1.4. | | | * | S | | | | B)b.C)a.1.4.*S | | | | | | | | |  |  |  |  |  |  |  |  |  |  |  |  |  |  |  |  |  |  |  |  |
| Berger,2011 | B)b.C)a. | | 1.4. | | | *# | S | | | | B)b.C)a.1.4.*#S | | | | | | | | |  |  |  |  |  |  |  |  |  |  |  |  |  |  |  |  |  |  |  |  |
| Bergström,2010 | B)b.c.C)a.D)b. | | 1.4. | | | *# | PG | | | | B)b.c.C)a.D)b.1.4.*#PG | | | | | | | | |  |  |  |  |  |  |  |  |  |  |  |  |  |  |  |  |  |  |  |  |
| Berry,2014 | B)b.D)b. | | 1. | | | *# | PG | | | | B)b.D)b.1.*#PG | | | | | | | | |  |  |  |  |  |  |  |  |  |  |  |  |  |  |  |  |  |  |  |  |
| Boele,2018 | C)a. | | 1. | | | # | PG | | | | C)a.1.#PG | | | | | | | | |  |  |  |  |  |  |  |  |  |  |  |  |  |  |  |  |  |  |  |  |
| Bond,2010 | B)a.b.c.C)b.D)b. | | 1. | | | *# | PG | | | | B)a.b.c.C)b.D)b.1.*#PG | | | | | | | | |  |  |  |  |  |  |  |  |  |  |  |  |  |  |  |  |  |  |  |  |
| Børøsund,2014 | A)a.B)b. | | 1.4. | | | # | PG | | | | A)a.B)b.1.4.#PG | | | | | | | | |  |  |  |  |  |  |  |  |  |  |  |  |  |  |  |  |  |  |  |  |
| Bowler,2012 | C)a. | | 2. | | | * | S | | | | C)a.2.*S | | | | | | | | |  |  |  |  |  |  |  |  |  |  |  |  |  |  |  |  |  |  |  |  |
| Braamse,2015 | C)b. | | 1. | | | # | S | | | | C)b.1.#S | | | | | | | | |  |  |  |  |  |  |  |  |  |  |  |  |  |  |  |  |  |  |  |  |
| Bromberg,2012 | B)a.C)a. | | 1. | | | # | PG | | | | B)a.C)a.1.#PG | | | | | | | | |  |  |  |  |  |  |  |  |  |  |  |  |  |  |  |  |  |  |  |  |
| Buhrman,2013 | C)a. | | 1. | | | # | S | | | | C)a.1.#S | | | | | | | | |  |  |  |  |  |  |  |  |  |  |  |  |  |  |  |  |  |  |  |  |
| Buhrman,2011 | C)a. | | 1. | | | * | S | | | | C)a.1.*S | | | | | | | | |  |  |  |  |  |  |  |  |  |  |  |  |  |  |  |  |  |  |  |  |
| Buhrman,2013 | C)b. | | 1. | | | # | S | | | | C)b.1.#S | | | | | | | | |  |  |  |  |  |  |  |  |  |  |  |  |  |  |  |  |  |  |  |  |
| Buhrman,2015 | C)a. | | 1. | | | # | S | | | | C)a.1.#S | | | | | | | | |  |  |  |  |  |  |  |  |  |  |  |  |  |  |  |  |  |  |  |  |
| Carlbring,2011 | C)a. | | 1. | | | # | S | | | | C)a.1.#S | | | | | | | | |  |  |  |  |  |  |  |  |  |  |  |  |  |  |  |  |  |  |  |  |
| Carpenter,2014 | B)a.C)a. | | 1. | | | *# | S | | | | B)a.C)a.1.*#S | | | | | | | | |  |  |  |  |  |  |  |  |  |  |  |  |  |  |  |  |  |  |  |  |
| Carrard,2011 | C)a. | | 1. | | | # | S | | | | C)a.1.#S | | | | | | | | |  |  |  |  |  |  |  |  |  |  |  |  |  |  |  |  |  |  |  |  |
| Classen,2012 | B)b.C)b. | | 1. | | | # | PG | | | | B)b.C)b.1.#PG | | | | | | | | |  |  |  |  |  |  |  |  |  |  |  |  |  |  |  |  |  |  |  |  |
| Cohn,2014 | C)b. | | 1. | | | # | PG | | | | C)b.1.#PG | | | | | | | | |  |  |  |  |  |  |  |  |  |  |  |  |  |  |  |  |  |  |  |  |
| Cooper,2011 | C)a. | | 2. | | | # | S | | | | C)a.2.#S | | | | | | | | |  |  |  |  |  |  |  |  |  |  |  |  |  |  |  |  |  |  |  |  |
| Damholdt,2016 | B)a. | | 1.4. | | | *# | S | | | | B)a.1.4.*#S | | | | | | | | |  |  |  |  |  |  |  |  |  |  |  |  |  |  |  |  |  |  |  |  |
| David,2012 | B)b.C)a. | | 1.4. | | | # | PG | | | | B)b.C)a.1.4.#PG | | | | | | | | |  |  |  |  |  |  |  |  |  |  |  |  |  |  |  |  |  |  |  |  |
| Davis,2013 | C)b. | | 1. | | | # | S | | | | C)b.1.#S | | | | | | | | |  |  |  |  |  |  |  |  |  |  |  |  |  |  |  |  |  |  |  |  |
| Dear,2015 | B)b.C)a. | | 1.4. | | | *# | S | | | | B)b.C)a.1.4.*#S | | | | | | | | |  |  |  |  |  |  |  |  |  |  |  |  |  |  |  |  |  |  |  |  |
| Dear,2013 | C)a. | | 1.4. | | | *# | PG | | | | C)a.1.4.*#PG | | | | | | | | |  |  |  |  |  |  |  |  |  |  |  |  |  |  |  |  |  |  |  |  |
| Devi,2014 | B)a.b.Db. | | 1.4. | | | # | S | | | | B)a.b.Db.1.4.#S | | | | | | | | |  |  |  |  |  |  |  |  |  |  |  |  |  |  |  |  |  |  |  |  |
| Dowd,2015 | C)a. | | 1.4. | | | # | S | | | | C)a.1.4.#S | | | | | | | | |  |  |  |  |  |  |  |  |  |  |  |  |  |  |  |  |  |  |  |  |
| Drozd,2014 | C)b. | | 1.4. | | | # | S | | | | C)b.1.4.#S | | | | | | | | |  |  |  |  |  |  |  |  |  |  |  |  |  |  |  |  |  |  |  |  |
| Engel,2015 | B)a.C)b.D)b. | | 1. | | | # | PG | | | | B)a.C)b.D)b.1.#PG | | | | | | | | |  |  |  |  |  |  |  |  |  |  |  |  |  |  |  |  |  |  |  |  |
| Everitt,2013 | C)a. | | 1. | | | * | S | | | | C)a.1.*S | | | | | | | | |  |  |  |  |  |  |  |  |  |  |  |  |  |  |  |  |  |  |  |  |
| Farrer,2011 | B)b.C)a. | | 1. | | | # | PG | | | | B)b.C)a.1.#PG | | | | | | | | |  |  |  |  |  |  |  |  |  |  |  |  |  |  |  |  |  |  |  |  |
| Freeman,2015 | 6. | | | | | * | N/A | | | C)b.D)b.6. | | | | |  |  |  |  |  |  |  |  |  |  |  |  |  |  |  |  |  |  |  |  |  |  |  |  |  |
| Friesen,2017 | C)a. | | 1.4. | | | # | | | S | | | | C)a.1.4.#S | | | | | | | | |  | | | | | | |  | | | | |  | |  | | | |
| Gerhards,2010 | C)a. | | 1. | | | # | | | S | | | | C)a.1.#S | | | | | | | | |  | | | | | | |  | | | | |  | |  | | | |
| Glozier,2013 | C)a. | | 1. | | | # | | | S | | | | C)a.1.#S | | | | | | | | |  | | | | | | |  | | | | |  | |  | | | |
| Hedborg,2011 | C)d. | | 1.4. | | | # | | | S | | | | C)d.1.4.#S | | | | | | | | |  | | | | | | |  | | | | |  | |  | | | |
| Hedman,2012 | B)a.b.C)a. | | 1. | | | # | | | S | | | | B)a.b.C)a.1.#S | | | | | | | | |  | | | | | | |  | | | | |  | |  | | | |
| Hedman,2014 | B)b.C)a. | | 1. | | | * | | | S | | | | B)b.C)a.1.*S | | | | | | | | |  | | | | | | |  | | | | |  | |  | | | |
| Hedman,2011 | B)a.b.c.C)a. | | 1.6. | | | # | | | PG | | | | B)a.b.c.C)a.1.6.#PG | | | | | | | | |  | | | | | | |  | | | | |  | |  | | | |
| Hesser,2012 | B)b.C)a. | | 1. | | | # | | | PG | | | | B)b.C)a.1.#PG | | | | | | | | |  | | | | | | |  | | | | |  | |  | | | |
| Høybye,2010 | B)c. | | 1. | | | * | | | B)c.1.* | | | | | | | | |  | | | | | | |  | |  | | | | |  | | | | | |  |  |
| Ivarsson,2014 | B)a.C)a. | | 1. | | | # | | | S | | | | B)a.C)a.1.#S | | | | | | | | |  | | | | | | |  | | | | |  | |  | | | |
| Jacobi,2012 | B)b.c.C)a.D)a.b. | | 1.4. | | | *# | | | PG | | | | B)b.c.C)a.D)a.b.1.4.*#PG | | | | | | | | |  | | | | | | |  | | | | |  | |  | | | |
| Jasper,2014 | C)a. | | 1.4. | | | # | | | PG | | | | C)a.1.4.#PG | | | | | | | | |  | | | | | | |  | | | | |  | |  | | | |
| Johansson,2015 | B)b.c.C)d. | | 1.6. | | | * | | | G | | | | B)b.c.C)d.1.6.*G | | | | | | | | |  | | | | | | |  | | | | |  | |  | | | |
| Johnston,2011 | B)b.C)a. | | 1.4. | | | *# | | | PG | | | | B)b.C)a.1.4.*#PG | | | | | | | | |  | | | | | | |  | | | | |  | |  | | | |
| Knaevelsrud,2015 | B)b.C)a.D)a. | | 1.4.6. | | | *# | | | PG | | | | B)b.C)a.D)a.1.4.6.*#PG | | | | | | | | |  | | | | | | |  | | | | |  | |  | | | |
| Kok,2014 | C)a. | | 1. | | | # | | | S | | | | C)a.1.#S | | | | | | | | |  | | | | | | |  | | | | |  | |  | | | |
| Kraaij,2010 | C)a.D)b. | | 1.2.10. | | | # | | | S | | | | C)a.D)b.1.2.10. #S | | | | | | | | |  | | | | | | |  | | | | |  | |  | | | |
| Kristjánsdóttir,2013 | B)b.C)a. | | 1.3. | | | *# | | | PG | | | | B)b.C)a.1.3.*#PG | | | | | | | | |  | | | | | | |  | | | | |  | |  | | | |
| Krupnick,2017 | C)b. | | 1. | | | # | | | PG | | | | C)b.1.#PG | | | | | | | | |  | | | | | | |  | | | | |  | |  | | | |
| Kuhn,2017 | C)a. | | 3. | | | # | | | S | | | | C)a.3.#S | | | | | | | | |  | | | | | | |  | | | | |  | |  | | | |
| Lepore,2014 | B)c. | | 1.4. | | | *# | | | PG | | | | B)c.1.4.*#PG | | | | | | | | |  | | | | | | |  | | | | |  | |  | | | |
| Lewis,2017 | C)a. | | 1.4. | | | # | | | PG | | | | C)a.1.4.#PG | | | | | | | | |  | | | | | | |  | | | | |  | |  | | | |
| Littleton,2016 | C)a. | | 1. | | | *# | | | G | | | | C)a.1.*#G | | | | | | | | |  | | | | | | |  | | | | |  | |  | | | |
| Ljótsson,2011 | C)a. | | 1.4. | | | # | | | PG | | | | C)a.1.4.#PG | | | | | | | | |  | | | | | | |  | | | | |  | |  | | | |
| Ljótsson,2010 | C)a. | | 1. | | | # | | | S | | | | C)a.1.#S | | | | | | | | |  | | | | | | |  | | | | |  | |  | | | |
| Lorig,2010 | C)b. | | 1. | | | # | | | S | | | | C)b.1.#S | | | | | | | | |  | | | | | | |  | | | | |  | |  | | | |
| Lundgren,2016 | C)a. | | 1.4. | | | # | | | PG | | | | C)a.1.4.#PG | | | | | | | | |  | | | | | | |  | | | | |  | |  | | | |
| Mailey,2010 | C)b.D)b. | | | 2. | | | | | PG | | | | | C)b.D)b.2.#PG | |  | | |  | | | |  | | | | | | |  |  |  |  |  |  |  |  |  |  |
| Matthews,2011 | C)b. | 2. | | | *# | | | S | | | | C)b.2.*#S | | | | | | | | |  | | | | | | |  | | | | |  | |  | | | |  |
| Migliorini,2016 | C)a. | 1. | | | # | | | S | | | | C)a.1.#S | | | | | | | | |  | | | | | | |  | | | | |  | |  | | | |  |
| Miner,2016 | C)a. | 3.4. | | | # | | | S | | | | C)a.3.4.#S | | | | | | | | |  | | | | | | |  | | | | |  | |  | | | |  |
| Newby,2013 | C)a. | 1. | | | # | | | S | | | | C)a.1.#S | | | | | | | | |  | | | | | | |  | | | | |  | |  | | | |  |
| Newby,2014 | C)a. | 1.4. | | | # | | | PG | | | | C)a.1.4.#PG | | | | | | | | |  | | | | | | |  | | | | |  | |  | | | |  |
| Newby,2017 | C)a. | 1.4. | | | # | | | PG | | | | C)a.1.4.#PG | | | | | | | | |  | | | | | | |  | | | | |  | |  | | | |  |
| Nordgren,2014 | C)a. | 1.4. | | | # | | | PG | | | | C)a.1.4.#PG | | | | | | | | |  | | | | | | |  | | | | |  | |  | | | |  |
| Osei,2013 | B)c. | 1. | | | # | | | S | | | | B)c.1.#S | | | | | | | | |  | | | | | | |  | | | | |  | |  | | | |  |
| Paxling,2011 | C)a. | 1. | | | # | | | S | | | | C)a.1.#S | | | | | | | | |  | | | | | | |  | | | | |  | |  | | | |  |
| Peters,2017 | C)a.b. | 1. | | | # | | | S | | | | C)a.b.1.#S | | | | | | | | |  | | | | | | |  | | | | |  | |  | | | |  |
| Possemato,2011 | C)b. | 1. | | | # | | | PG | | | | C)b.1.#PG | | | | | | | | |  | | | | | | |  | | | | |  | |  | | | |  |
| Possemato,2015 | C)a. | 1. | | | # | | | PG | | | | C)a.1.#PG | | | | | | | | |  | | | | | | |  | | | | |  | |  | | | |  |
| Robinson,2010 | C)a. | 1.4. | | | # | | | PG | | | | C)a.1.4.#PG | | | | | | | | |  | | | | | | |  | | | | |  | |  | | | |  |
| Rosmarin,2010 | C)b. | 1. | | | # | | | S | | | | C)b.1.#S | | | | | | | | |  | | | | | | |  | | | | |  | |  | | | |  |
| Roy-Byrne,2010 | C)a.D)b. | 1. | | | # | | | S | | | | C)a.D)b.1.#S | | | | | | | | |  | | | | | | |  | | | | |  | |  | | | |  |
| Ruehlman,2012 | C)b.D)b. | 1. | | | # | | | S | | | | C)b.D)b.1.#S | | | | | | | | |  | | | | | | |  | | | | |  | |  | | | |  |
| Ruland,2013 | B)a.b.D)b. | 1. | | | # | | | PG | | | | B)a.b.D)b.1.#PG | | | | | | | | |  | | | | | | |  | | | | |  | |  | | | |  |
| Ruwaard,2010 | C)a. | 1. | | | *# | | | PG | | | | C)a.1.*#PG | | | | | | | | |  | | | | | | |  | | | | |  | |  | | | |  |
| Salzer,2009/2010 | B)c. | 1. | | |  | | | B)c.1. | | | | | | | | |  | | | | | | |  | |  | | | | |  | | | | | |  |  |  |
| Sanchez-Ortiz,2011 | C)a. | 1.4. | | | # | | | PG | | | | C)a.1.4.#PG | | | | | | | | |  | | | | | | |  | | | | |  | |  | | | |  |
| Seekles,2011 | B)a.b.c.C)b.D)b. | 1.6. | | | *# | | | PG | | | | B)a.b.c.C)b.D)b.1.6.*#PG | | | | | | | | |  | | | | | | |  | | | | |  | |  | | | |  |
| Sexton,2010 | C)a. | 1. | | | # | | | S | | | | C)a.1.#S | | | | | | | | |  | | | | | | |  | | | | |  | |  | | | |  |
| Shigaki,2013 | B)a.c.C)a. | 1. | | | # | | | S | | | | B)a.c.C)a.1.#S | | | | | | | | |  | | | | | | |  | | | | |  | |  | | | |  |
| Silfvernagel,2012 | C)a. | 1.4. | | | # | | | S | | | | C)a.1.4.#S | | | | | | | | |  | | | | | | |  | | | | |  | |  | | | |  |
| Spence,2014 | C)a. | 1.4. | | | # | | | S | | | | C)a.1.4.#S | | | | | | | | |  | | | | | | |  | | | | |  | |  | | | |  |
| Spence,2011 | B)b.c.C)a. | 1. | | | *# | | | PG | | | | B)b.c.C)a.1.*#PG | | | | | | | | |  | | | | | | |  | | | | |  | |  | | | |  |
| Stanton,2013 | B)a.b.c. | 1. | | | # | | | S | | | | B)a.b.c.1.#S | | | | | | | | |  | | | | | | |  | | | | |  | |  | | | |  |
| Thompson,2015 | C)a. | 1. | | | # | | | S | | | | C)a.1.#S | | | | | | | | |  | | | | | | |  | | | | |  | |  | | | |  |
| Thompson,2010 | B)b.C)a. | 1. | | | *# | | | PG | | | | B)b.C)a.1.*#PG | | | | | | | | |  | | | | | | |  | | | | |  | |  | | | |  |
| Titov,2010 | B)a.b.c.C)a. | 1.4. | | | *# | | | PG | | | | B)a.b.c.C)a.1.4.*#PG | | | | | | | | |  | | | | | | |  | | | | |  | |  | | | |  |
| Titov,2010 | B)a.b.c.C)a. | 1.4. | | | # | | | PG | | | | B)a.b.c.C)a.1.4.#PG | | | | | | | | |  | | | | | | |  | | | | |  | |  | | | |  |
| Trompetter,2014 | C)a.b. | 1. | | | # | | | PG | | | | C)a.b.1.#PG | | | | | | | | |  | | | | | | |  | | | | |  | |  | | | |  |
| Trudeau,2015 | B)b.C)a.D)b. | 1. | | | # | | | PG | | | | B)b.C)a.D)b.1.#PG | | | | | | | | |  | | | | | | |  | | | | |  | |  | | | |  |
| Vallejo,2015 | B)a.C)a. | 1. | | | # | | | PG | | | | B)a.C)a.1.#PG | | | | | | | | |  | | | | | | |  | | | | |  | |  | | | |  |
| van Ballegooijen,2013 | B)b.c.C)a. | 1. | | | # | | | PG | | | | B)b.c.C)a.1.#PG | | | | | | | | |  | | | | | | |  | | | | |  | |  | | | |  |
| van Bastelaar,2011 | B)b.C)a. | 1. | | | # | | | PG | | | | B)b.C)a.1.#PG | | | | | | | | |  | | | | | | |  | | | | |  | |  | | | |  |
| van den Berg,2015 | C)a.D)b. | 1. | | | * | | | S | | | | C)a.D)b.1.*S | | | | | | | | |  | | | | | | |  | | | | |  | |  | | | |  |
| Varley,2011 | C)d. | 1. | | | # | | | S | | | | C)d.1.#S | | | | | | | | |  | | | | | | |  | | | | |  | |  | | | |  |
| Vernmark,2010 | B)b.C)a. | 1.4. | | | # | | | S | | | | B)b.C)a.1.4.#S | | | | | | | | |  | | | | | | |  | | | | |  | |  | | | |  |
| Weinert,2011 | B)c.C)d. | 1. | | | # | | | S | | | | B)c.C)d.1.#S | | | | | | | | |  | | | | | | |  | | | | |  | |  | | | |  |
| Weise,2016 | B)b.C)a. | 1. | | | # | | | S | | | | B)b.C)a.1.#S | | | | | | | | |  | | | | | | |  | | | | |  | |  | | | |  |
| Willems,2017 | C)a.b. | 1. | | | # | | | S | | | | C)a.b.1.#S | | | | | | | | |  | | | | | | |  | | | | |  | |  | | | |  |
| Williams,2010 | C)a.D)b. | 1. | | | # | | | S | | | | C)a.D)b.1.#S | | | | | | | | |  | | | | | | |  | | | | |  | |  | | | |  |
| Wilson,2014 | C)d. | 1. | | | # | | | S | | | | C)d.1.#S | | | | | | | | |  | | | | | | |  | | | | |  | |  | | | |  |
| Wilson,2017 | C)a. | 1. | | | # | | | S | | | | C)a.1.#S | | | | | | | | |  | | | | | | |  | | | | |  | |  | | | |  |
| Wims,2010 | B)a.b.c.C)a. | 1.4. | | | # | | | PG | | | | B)a.b.c.C)a.1.4.#PG | | | | | | | | |  | | | | | | |  | | | | |  | |  | | | |  |
| Wootten,2015 | C)a. | 1. | | | # | | | S | | | | C)a.1.#S | | | | | | | | |  | | | | | | |  | | | | |  | |  | | | |  |
| Xie,2013 | C)a. | 1. | | | # | | | PG | | | | C)a.1.#PG | | | | | | | | |  | | | | | | |  | | | | |  | |  | | | |  |
| Yanez,2015 | B)b.c.C)a.D)b. | 1. | | | *# | | | PG | | | | B)b.c.C)a.D)b.1.*#PG | | | | | | | | |  | | | | | | |  | | | | |  | |  | | | |  |
| Younge,2015 | A)b.C)b. | 1.4. | | | # | | | S | | | | A)b.C)b.1.4.#S | | | | | | | | |  | | | | | | |  | | | | |  | |  | | | |  |
| Yun,2012 | A)b.B)a.C)a.D)a.b. | 1. | | | *# | | | S | | | | A)b.B)a.C)a.D)a.b.1.*#S | | | | | | | | |  | | | | | | |  | | | | |  | |  | | | |  |
| Zernicke,2014 | C)b. | 1. | | | * | | | PG | | | | C)b.1.*PG | | | | | | | | |  | | | | | | |  | | | | |  | |  | | | |  |

*System:* **1.** Internet or Website **2.** Computer (software) **3.** Mobile app **4.** Electronic messaging (email, SMS) **5.** Electronic health record **6.** Telehealth (telemedicine, telepsychiatry) **7.** Virtual reality/ augmented reality **8.** Robot **9.** Connected devices **10.** Social media **11.** Other system;

*Function:* **A.**Decision support **a)**Screening **b)**Prompts and alerts **B.** Communication **a.** Transmission of information (one way) **b.** Communication (with healthcare provider) **c.** Communication (peer to peer, e.g., virtual peer group for clients) **C.** Therapy **a.** Cognitive Behavioural Therapy (CBT) **b.** Other psychotherapy **c.** Gamification **D.** Monitoring **a.** Provider monitoring **b.** Self-monitoring;

*Time:* **=** Synchronous **+** Asynchronous

*Facilitation:* ***G*.** Entirely supported by healthcare providers ***PG****.*Partially supported by healthcare providers **S.** Self-administered
