# Supplementary material for: The impact of technology systems and level of support in digital mental health interventions: a secondary meta-analysis
Source: Syst Rev. 2023 May 4;12:78. doi: 10.1186/s13643-023-02241-1 (PMC10157597; doi:10.1186/s13643-023-02241-1)
Supplement: Supplementary file 7 — Additional file 7. Forest plot: Subgroup analysis on type of technology for any digital intervention vs. usual care or another digital intervention to manage depression in people with any concomitant chronic condition. [file 13643_2023_2241_MOESM7_ESM.docx]

Additional file 7. *Forest plot: Subgroup analysis on type of technology for any digital intervention vs. usual care or another digital intervention to manage depression in people with any concomitant chronic condition.*
